# Assessment of dysplasia in bone marrow smear with convolutional neural network

**DOI:** 10.1038/s41598-020-71752-x

**Published:** 2020-09-07

**Authors:** Jinichi Mori, Shizuo Kaji, Hiroki Kawai, Satoshi Kida, Masaharu Tsubokura, Masahiko Fukatsu, Kayo Harada, Hideyoshi Noji, Takayuki Ikezoe, Tomoya Maeda, Akira Matsuda

**Affiliations:** 1 Department of Hematology, Jyoban Hospital, Tokiwa Foundation, 57 Jyoban kamiyunagayamachi kaminodai, Iwaki, Fukushima 972-8322 Japan; 2 Institute of Mathematics for Industry, Kyushu University, Fukuoka, Japan; 3 Research and Development Department, LPIXEL Inc., Tokyo, Japan; 4 Department of Public Health, Fukushima Medical University, Fukushima, Japan; 5 Department of Hematology, Fukushima Medical University, Fukushima, Japan; 6 Department of Hematology, Minami Fukushima Cardiovascular Hospital, Fukushima, Japan; 7 Department of Hemato-Oncology, International Medical Center, Saitama Medical University, Saitama, Japan

**Keywords:** Myelodysplastic syndrome, Image processing, Machine learning

## Abstract

In this study, we developed the world's first artificial intelligence (AI) system that assesses the dysplasia of blood cells on bone marrow smears and presents the result of AI prediction for one of the most representative dysplasia—decreased granules (DG). We photographed field images from the bone marrow smears from patients with myelodysplastic syndrome (MDS) or non-MDS diseases and cropped each cell using an originally developed cell detector. Two morphologists labelled each cell. The degree of dysplasia was evaluated on a four-point scale: 0–3 (e.g., neutrophil with severely decreased granules were labelled DG3). We then constructed the classifier from the dataset of labelled images. The detector and classifier were based on a deep neural network pre-trained with natural images. We obtained 1797 labelled images, and the morphologists determined 134 DGs (DG1: 46, DG2: 77, DG3: 11). Subsequently, we performed a five-fold cross-validation to evaluate the performance of the classifier. For DG1–3 labelled by morphologists, the sensitivity, specificity, positive predictive value (PPV), negative predictive value (NPV), and accuracy were 91.0%, 97.7%, 76.3%, 99.3%, and 97.2%, respectively. When DG1 was excluded in the process, the sensitivity, specificity, PPV, NPV, and accuracy were 85.2%, 98.9%, 80.6%, and 99.2% and 98.2%, respectively.

## Introduction

Many attempts have been made in the past decade to automatically determine cell types in blood smears. Initially, researchers developed algorithms to detect leukocytes, red blood cells, or nuclear segmentation^1–10^. Subsequently, they started addressing the detection of abnormal leukocytes including various types of leukemic cells^11–17^. However, these works mainly focused on peripheral blood smears, and few studies have covered bone marrow due to their greater complexity^18–20^. As there are many types of progenitor cells with various stages of continuous maturation in bone marrow specimens, a microscopic field contains a larger amount of information compared to peripheral blood. Moreover, the examination of bone marrow smears requires morphological evaluation in clinical settings, whereas the examination of peripheral blood mainly focuses on cell counting. These hurdles have prevented the development of automated examination of bone marrow smears and delayed the application of machine learning technology in the diagnosis of bone marrow disorders.

Myelodysplastic syndrome (MDS) is a haematological disease that develops mainly in the elderly and is characterised by an abnormal morphology (dysplasia) of blood cells in bone marrow. Haematopoietic progenitor cells, which acquire certain somatic gene mutations, clonally expand in bone marrow, leading to cytopenia characterised by ineffective haematopoiesis with myelodysplasia. Progressive cytopenia in multiple blood lineages and transformation to acute myeloid leukaemia are causes of death in patients with MDS. The morphological examination of bone marrow smears using light microscopy plays a critical role in the diagnosis of MDS. Since the first report of this disease, various types of dysplasia in cell lineages have been identified, such as granulocyte, erythrocyte, and megakaryocyte. The presence of bone marrow dysplasia, which is a requisite condition for the diagnosis of MDS, is defined as 10% or more of dysplastic cells in each cell lineage, or 5–20% of myeloblasts in all nucleated cells^21^.

Notably, expert skill in morphological examination is required for the accurate diagnosis of dysplasia in bone marrow smears; however, such expert review is not always available in daily clinical practice. Therefore, it is necessary to develop an artificial intelligence (AI) system that assists with the morphological assessment of dysplasia in bone marrow smears, but no reports exist in this field to date. In the current study, we developed an AI system that can diagnose ‘decreased granules (DG)’ in neutrophil, one of the most representative forms of dysplasia.

## Materials and methods

### Data collection

**Figure 1:**
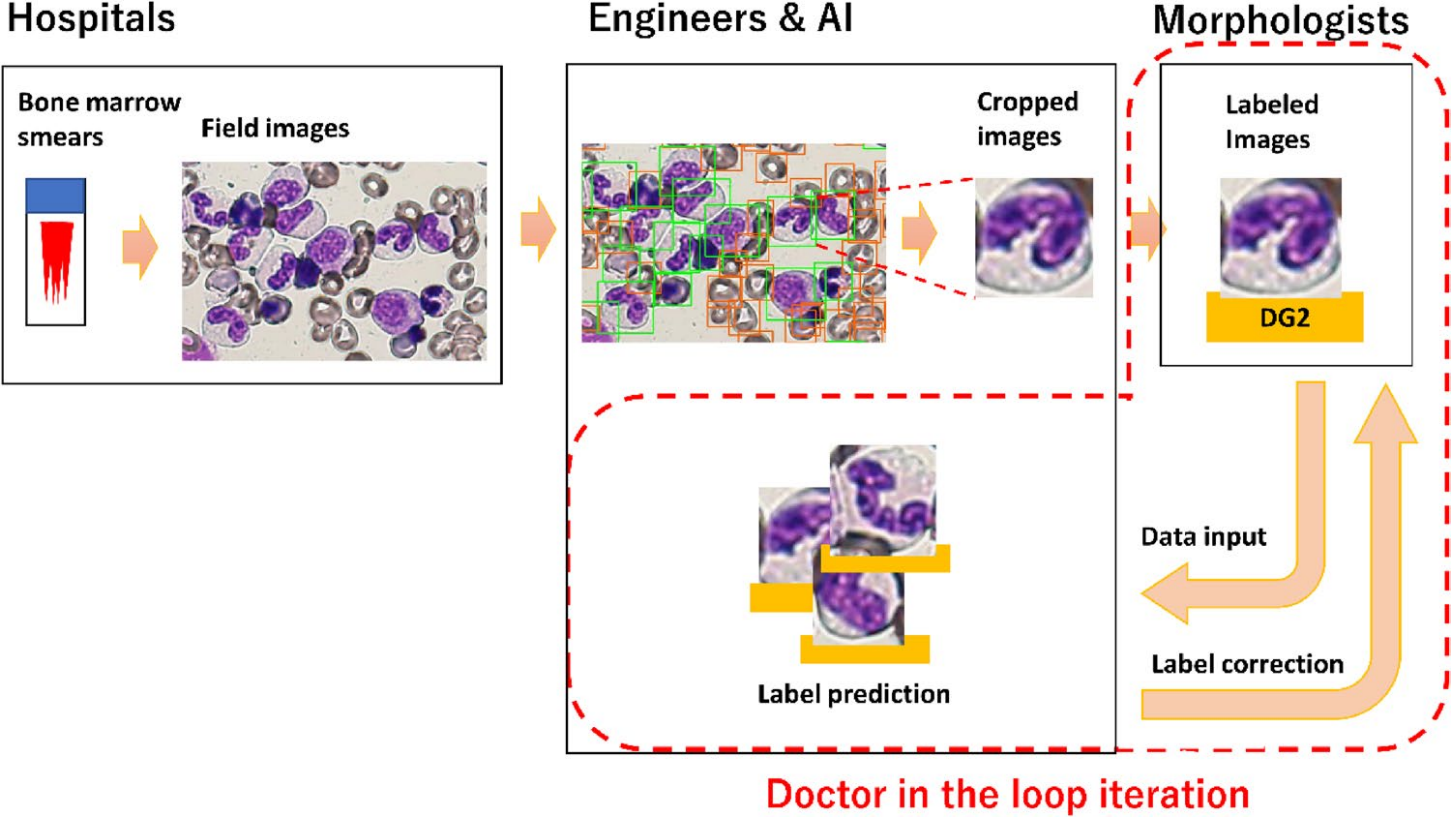
Workflow of the AI construction. Microscopic images from bone marrow smears in hospitals were digitalised into field images. Each single cell was cropped by the originally developed detector. The morphologists labelled them, and these labelled images were fed into the regressor. The AI system’s predictions were presented back to the morphologists for re-evaluation of the labels (Doctor in the loop).

**Figure 2:**
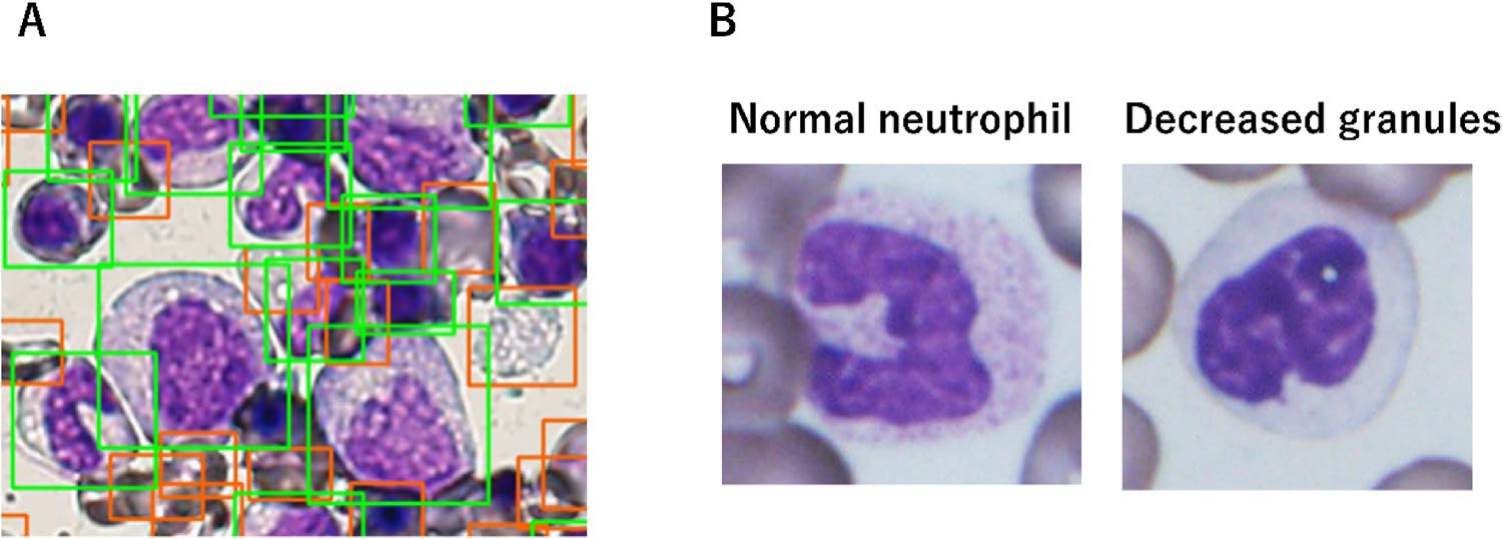
Detector and targeted dysplasia. (**A**) The detector automatically extracts the single cells from the field images. It distinguishes between the subjects that are of interest (green boxes: nucleated cells) and those that are not (orange boxes: red blood cells, platelets or trashes). (**B**) Normal neutrophils (left). Decreased granules; pink and fine granules in the cytoplasm are markedly reduced in the neutrophil with decreased granules (right).

Figure 1 illustrates the workflow of the AI construction. We collected bone marrow smears stained with May–Grunwald–Giemsa stain from patients who had been diagnosed with MDS or other haematological disease (to obtain images of normal blood cells) at Jyoban Hospital and Fukushima Medical University Hospital between 2011–2018. Two clinicians photographed field images of the bone marrow smears at 100 times magnification using cellSens Standard (version 2.1). Each cell in the field images was detected using the Tensorflow Object Detection API (https://github.com/tensorflow/models/tree/master/research/object_detection), and they were cropped into rectangular images such that each cropped image contained only a single cell—provided the cells did not overlap (Fig. 2A). We trained Faster R-CNN with ResNet-101 backbone using a part of the images mentioned above. All blood cells were annotated with bounding boxes, which were respectively tagged as such. We used a repository’s sample configuration for COCO dataset with only minor changes to num_classes, and max/min_dimention. The ratio of second_stage_classification_loss_weight to second_stage_classification_loss_weight was set to 2.0. All the field images were then passed to the trained Faster R-CNN. Output annotations that were wrong (e.g., the ones containing two cells) were corrected by a human annotator such that each of the boxes contained a single cell. Subsequently, each cell was cropped into a rectangular image in preparation for the labelling process.

Two experts (AM and TM) in blood cell morphology labelled each cell in the cropped image by discussing with each other. We first excluded squashed, broken, or out-of-focus cells, as well as dust and bare nuclei. Next, we divided the cells into ‘applicable’ and ‘not applicable’ for the determination of dysplasia. Dysplasia was defined according to the World Health Organisation Classification of Tumours of Haematopoietic and Lymphoid Tissues, revised, 4th edition (2017), and we added some factors defined by the International Working Group on Morphology of Myelodysplastic Syndrome classification (Supplementary Table 1)^22^. The degree of dysplasia was evaluated on a four-point scale—0: normal; 1: intermediate, i.e., between normal and dysplasia; 2: dysplasia; and 3: severe dysplasia. As there is no universal grading system for dysplasia, our grading depended on the morphologists. We refer to a pair of the single-cell image and its label as the *labelled image*. Among the various types of dysplasia, we focused on DG in neutrophils (Fig. 2B) because it is regarded as a highly representative dysplasia for which we had relatively abundant labelled images.

**Table 1:** Confirmed labels by morphologists.

	Abbreviations	Number of cells		
Applicable cells				
Erythrocytes				
Normal erythroid cells	NE	392		
		Scale of dysplasia		
Dysplastic erythroid cells		1	2	3
Nuclear budding	NB	5	21	4
Internuclear bridging	INB	0	0	2
Karyorrhexis	KR	0	3	2
Multinuclearity	MN	0	2	5
Red cell abnormal chromatin clamping	RCACC	64	30	5
Megaloblastoid change	MC	2	0	0
Giant red cell	GRC	4	13	9
Vacuolization	VAC	0	2	0
Howell–Jolly bodies	HJB	0	1	1
Granulocytes				
Normal neutrophils	NN	96		
Myeloblasts	MB	62		
		Scale of dysplasia		
Dysplastic granulocytes		1	2	3
Small size or unusually large size	SUL	0	0	0
Nuclear hyposegmentation (Psudo-Pelger–Huët)	HS	4	12	22
Nuclear hypersegmentation	HYPES	1	1	0
Decreased granules; agranularity	DG	46	77	11
Pseudo-Chédiak–Higashi granules	PCH	0	0	0
Döhle bodies	DB	0	0	0
Auer rods	AR	0	0	0
Dysplastic non-Psudo-Pelger–Huët	DNP	0	1	0
Nuclear projections	NP	7	8	0
Abnormal chromatin clumping	ACC	47	17	1
Megakaryocytes				
Normal megakaryocytes	NM	98		
		Scale of dysplasia		
Dysplastic megakaryocytes		1	2	3
Micromegakaryocytes	MM	1	0	1
Nuclear hypolobation	NH	29	20	11
Multinucleation	MN	5	7	5
Large megakaryocyte with a hyperlobulated nucleus	LM	1	1	0
Megakaryocytes with cytoplasmic abnormality	MCA	0	0	0
Not applicable cells				
Red blood cells	RBC	126		
Dividing erythrocytes	DE	6		
Immature granulocytes	IG	440		
Basophils	BAS	1		
Eosinophils	EOS	18		
Monocytes	MON	24		
Lymphocytes	LYM	78		
Plasma cells	PC	29		
Histiocytes	HIS	10		
Pletelets	PLT	29		
Mitotic cells	MIT	14		

Informed consent was obtained from all patients. This study was reviewed by the local ethics committees of Jyoban Hospital and Fukushima Medical University, and it was conducted according to the ethical principles of the Declaration of Helsinki.

### Dysplasia detection by convolutional neural networks

**Figure 3:**
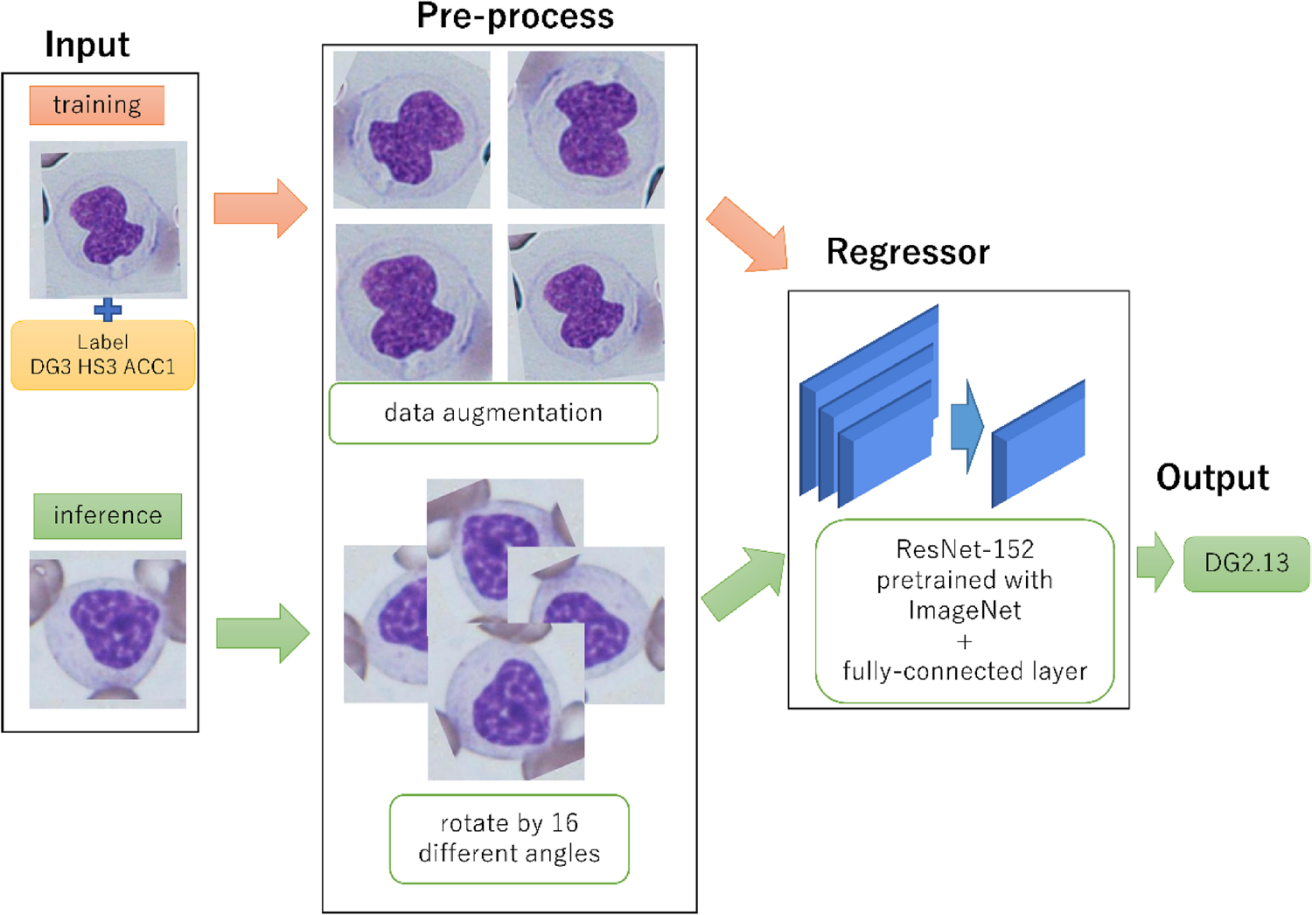
Structure and data flow of our system. Inference is performed in an ensemble manner by applying the trained regressor to different rotations of a single image.

We randomly divided the labelled images into five strata such that each stratum contained approximately the same number of images of each type of dysplasia. For the stratification, we used a publicly available python code (https://github.com/trent-b/iterative-stratification). We trained a regressor on four out of the five strata. The performance was measured using five-fold cross-validation on these five strata. As our dataset was relatively small, we utilized transfer learning. Our deep neural network-based regressor consisted of two parts: ResNet-152 truncated at the last pooling layer, followed by a fully connected linear layer with an output dimension of 34, which is the number of label types^23^. Our codes are available online (https://github.com/shizuo-kaji/MyelodysplasticSyndrome). ResNet-152 was pre-trained for image classification using ImageNet, which is a large dataset of natural images. Between the two parts, a dropout at the ration of 0.5 was applied. After the fully connected layer, sigmoid and scaling were applied. For example, if a label was scaled from null to four, the corresponding output was scaled by four. We also tested linear and ReLU outputs but observed deterioration in performance. The outputs of some samples were well outside the legitimate range, and they seemed to affect the loss optimisation. We used the weighted-mean-quartic loss for the loss function. To prioritise the low loss values for the focused label DG, the output corresponding to the focused label DG was weighted 10 times more. We tested a few other loss functions, including the mean-squared-error, contrastive loss, and focal loss. However, we found that the weighted-mean-quartic loss was the best choice because it penalises large deviation more (we did not want our AI system to make significant errors). We used a regression model and tested a classification model, replacing the final output layer with softmax. The performance worsened, which can be explained by the larger output dimension of the classification model required for the on-hot encoding of classes. We used Adam optimiser with a learning rate of 10-5 for the first (pretrained) part and 10-4 for the fully connected part. An L2 weighted regularization of 10-6 was used to prevent overfitting. Training was performed using 120 epochs with a batch size of 10, but we observed that the training converged after approximately 90 epochs. The main difficulty in training the regressor was that the dataset was highly imbalanced. For example, only 13 out of 1797 images were labelled as DG3. A standard strategy to deal with imbalanced data is to weigh the loss function. However, with our extreme imbalance, we found that data augmentation resulted in better performance. Before applying the data augmentation transformations, each labelled image was pasted in the centre of a large field image to remove boundary effects. For every epoch, each labelled image was randomly translated, rotated, flipped, and scaled by 0.8–1.2 before being fed into the regressor (Fig. 3). The images with rare labels were fed multiple times into an epoch such that the total number of images with each label became approximately the same. The inference was made in the following manner. First, each image was rotated by 22.5 × n degrees (n = 0, 1, …, 15) and fed into the regressor. The maximum score for each label among the 16 rotated images was then computed. The 34 output classes belonged to one of the four non-overlapping categories: ‘erythrocyte’, ‘granulocyte’, ‘megakaryocyte’, or ‘not applicable’. The images fell into categories other than ‘granulocyte’ were given a final DG score of 0. Otherwise, the final DG score was set as the maximum of the 16 output scores for the DG label, rounded to the nearest integer, which were 0, 1, 2, and 3. The score depended on the rotation angle of the input image. Therefore, considering the maximum score among the 16 rotations was necessary.

### Doctor-in-the-loop

When we investigated the output of our machine learning system, we found that some of the predictions that contradicted the doctors’ labels were correct. We realised that not only the AI, but also the doctors had made mistakes. Thus, we adopted the following strategy which we named the ‘Doctor in the loop’ iteration. In each iteration, the doctors were shown the list of misclassified images with raw outputs from the AI system. Then, the doctors assessed where the AI system or humans had made a mistake. Further, the doctors corrected the wrong labels of the previous iteration. The engineers tuned the model and its hyper-parameters, and the AI system was trained again. Over these iterations, both the quality of the data and machine learning method described in the previous section were improved. In our case, it took five iterations before convergence. For each iteration, training the AI system lasted several hours (times fivefold cross-validation), and the morphologists’ re-evaluation lasted several minutes. Tuning the network structure and hyper-parameters lasted longest in each iteration, sometimes up to a few days. In total, convergence took a few months. We named the AI system ‘AKIRA’ after one of the morphologists in our group.

### Evaluation metrics

Classification performance of *AKIRA* wa*s* assessed using the following metrics:

$$ {\text{sensitivity}} = \frac{{{\text{true positive}}}}{{{\text{positive label}}}} $$

$$ {\text{specificity}} = \frac{{{\text{true negative}}}}{{{\text{negative label}}}} $$

$$ {\text{positive predictive value}} = \frac{{{\text{true positive}}}}{{{\text{positive prediction}}}} $$

$$ {\text{negative predictive value}} = \frac{{{\text{true negative}}}}{{{\text{negative prediction}}}} $$

$$ {\text{accuracy}} = \frac{{{\text{true positive}} + {\text{true negative}}}}{{{\text{true positive}} + {\text{false positive}} + {\text{true negative}} + {\text{false negative}}}} $$

‘true positive’ and ‘true negative’ are the number of images for which both the human label and network prediction are DG or not DG, respectively. ‘false positive’ and ‘false negative’ are the number of images for which the human label was not DG and network prediction was DG, or the human label was DG and network prediction was not DG. ‘positive label’ and ‘negative label’ are the number of total images labelled with DG or not labelled with DG, respectively.

## Results

We included 35 smears in the study and obtained 1797 labelled images from 386 field images. A summary of labels by morphologists is shown in Table 1. In the initial labelling process, the morphologists determined 125 DGs (DG1: 47 DG2: 65 DG3: 13). However, through three loop cycles between the AI and morphologists, 134 cells were finally determined as DG (DG1: 46, DG2: 77, DG3: 11). Table 2 summarises the labelling of DGs by the morphologists and prediction by *AKIRA*. When DG1–3 are defined as positive, the sensitivity, specificity, positive predictive value, and negative predictive value were 91.0%, 97.7%, 76.3%, and 99.3%, respectively. The area under the curve (AUC) was 0.944, and accuracy of the system was 97.2%. For clinical use, it is important to determine more obvious dysplasia such as DG2–3 than vague dysplasia such as DG1. We excluded DG1 labels and divided DG1 predictions into DG0 or DG2 based on their prediction probabilities. As a result, the sensitivity, specificity, positive predictive value, and negative predictive value were 85.2%, 98.9%, 80.6%, and 99.2%, respectively. The AUC was 0.921, and accuracy was 98.2%.

**Table 2:** Prediction versus true label (confusion matrix).

		Prediction			
		DG0	DG1	DG2	DG3
True label	DG0	1623	32	5	1
	DG1	5	21	19	1
	DG2	6	16	50	5
	DG3	1	2	7	1

**Figure 4:**
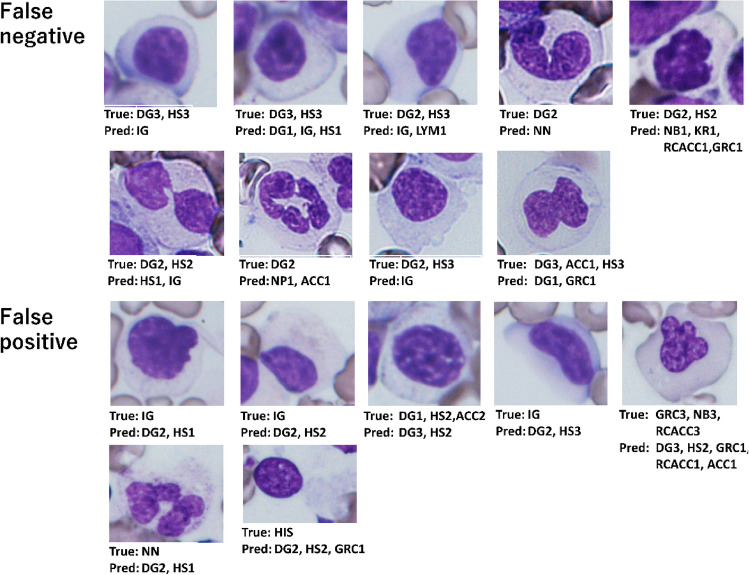
Images of the failed AI prediction. False positives are those predicted by the AI to be DG-positive, while labelling performed by humans is DG-negative. Conversely, false negatives are those predicted by the AI to be DG-negative, while labelling performed by humans is DG-positive. The list of label abbreviations is shown in Table 1.

To investigate the cause of DG misclassification, we extracted the false positive or false negative cells in which the discrepancy in scale between the label and prediction was two or more (e.g., the label was DG0, but the prediction was DG2 or DG3.) (Fig. 4). Six of the nine false negative cells were mistaken as immature granulocytes (IG), which resemble neutrophils with nuclear hyposegmentation (HS). Conversely, three of the seven false positive cells had a label of IG.

As dysplastic features in neutrophils are often associated with one another, it is possible that AKIRA dose not really recognise decreased granules but regards other dysplasia (e.g., HS, nuclear projection, and abnormal chromatin clamping) as DG. To address the possibility, we compared the accuracy of prediction between cells labelled as DG alone and those labelled as DG and HS—the most frequently associated with DG in our dataset. Among 168 cells with DG alone and 65 cells with DG and HS, AKIRA mis-predicted 7 (4%) and 15 (23%) cells with 2 or more scale differences, respectively. This result suggests that AKIRA might accurately learn DGs in the cytoplasm from the labelled images. If DG accompanies HS, they are often recognised as IG, which is excluded from dysplasia classification. This increases the false negative rate.

## Discussion

We have described an intelligent system that can determine a type of dysplasia in bone marrow smears, and to the best of our knowledge, this is the first study to do so. As noted above, the automated assessment of bone marrow specimen is highly challenging, and the determination of dysplasia is even more difficult. To overcome various hurdles, we devised a few techniques to specialise our system, with particular attention given to addressing the significant imbalance in the number of cells with specific labels. Although we have not conducted a validation study evaluating the concordance between the AI system and third-party morphologists, an accuracy of 97.2% is an outstanding result. Moreover, in excluding DG1, the accuracy was 98.2%. A similar previous study in which the convolutional neural network (CNN) was constructed using over 18,000 images of white blood cell showed that myeloblasts in blood smears were classified with a sensitivity of 94% (91.0% in our study) and a negative predictive value of 94% (99.3% in our study)^17^. It is notable that our CNN developed using far less images achieved such a high predictive performance. This high performance is attributable to our careful design of the loss functions and data augmentation scheme. To observe the effect of transfer learning, we trained the same model from scratch. The result is shown in Supplementary Table 2. The training (convergence) lasted three times longer (about 300 epochs compared to 100 epochs for transfer learning). The accuracy was 94.0% (against 97.2% for transfer learning), and the accuracy of DG2–3 was 95.7% (against 98.2% for transfer learning). Excluding DG1 enhanced the accuracy of the system, suggesting that obvious dysplasia is easier to classify for AI than ambiguous one as with humans. Since determining obvious dysplasia is important for diagnosis of MDS in clinical setting, our AI meets clinical requirements. There were very few (less than 1%) misclassifications with a high degree of discrepancy (scale difference ≥ 2) between the true label and that predicted by our system. In most of these misclassifications, *AKIRA* was unable to recognise the neutrophils due to another concomitant dysplasia, HS, which mimics immature granulocytes. This problem may be resolved when more data is accumulated and the determination accuracy of HS and IG are enhanced.

The accuracy of the AI system never exceeds that of humans as long as human labelling is the right answer. However, our AI system *AKIRA* assisted human judgement through three cycles of the ‘Doctor in the loop’ feedback process. Among the images whose initial labels differed from *AKIRA*’s prediction, the morphologists corrected over 10% of their labels. This indicates that *AKIRA* complemented human labelling and thereby enhanced its own accuracy. Such ‘human in the loop’ iteration methods are effective for AI systems that generate training data based on human judgement, as previously reported by another group^24^.

There are some limitations in the present study. First, we employed only two experts in the field morphology, and the review process was not independent. This might have biased the labelling of the cells. There is no gold standard for the morphological diagnosis of dysplasia. In particular, the diagnoses of dysplasia are not always concordant among morphologists^25^. However, a morphologist in our study was a member of the central reviewers in the Japanese National Research Group on Idiopathic Bone Marrow Failure Syndromes, in which the inter-observer agreement of this system is high^26^. This strengthened the labelling credibility in our study. Second, although our data augmentation method worked successfully for DG, our dataset included a small number of labelled images to predict other types of dysplasia. Thus, we intend to construct a larger dataset to develop a diagnostic AI system that covers a wide range of dysplasia types.

In this study, we developed the world’s first AI system that assesses a type of dysplasia of blood cells on bone marrow smears with a high predictive accuracy. Although many hurdles remain, we intend to develop an AI system that can diagnose MDS based on larger datasets and deploy it in real-world clinical practice.

## Supplementary information

Supplementary Tables.

## Data Availability

The source code is disclosed in https://github.com/shizuo-kaji/MyelodysplasticSyndrome. The datasets used for construction of AI in the current study are also available from the corresponding author on reasonable request.
