# Supplementary material for: Assessment of dysplasia in bone marrow smear with convolutional neural network
Source: Sci Rep. 2020 Sep 7;10:14734. doi: 10.1038/s41598-020-71752-x (PMC7477564; doi:10.1038/s41598-020-71752-x)
Supplement: Supplementary file 1 — Supplementary Tables. [file 41598_2020_71752_MOESM1_ESM.docx]

TITLE: Assessment of dysplasia in bone marrow smear with convolutional neural network

Jinichi Mori^1^*, Shizuo Kaji^2^, Hiroki Kawai^3^, Satoshi Kida^3^, Masaharu Tsubokura^4^, Masahiko Fukatsu^5^, Kayo Harada^5^, Hideyoshi Noji^5,6^, Takayuki Ikezoe^5^, Tomoya Maeda^7^ and Akira Matsuda^7^

1 Department of Hematology, Jyoban Hospital, Tokiwa Foundation, Fukushima, Japan

2 Institute of Mathematics for Industry, Kyushu University, Fukuoka, Japan

3 Research and Development Department, LPIXEL Inc., Tokyo, Japan

4 Department of Public Health, Fukushima Medical University, Fukushima, Japan

5 Department of Hematology, Fukushima Medical University, Fukushima, Japan

6 Department of Hematology, Minami Fukushima Cardiovascular Hospital, Fukushim
a, Japan

7 Department of Hemato-Oncology, International Medical Center, Saitama Medical
 University

Supplementary Table 1 Definition of dysplasia

| Dyserythropoiesis |
| --- |
| Nuclear budding |
| Internuclear bridging |
| Karyorrhexis |
| Multinuclearity |
| Red cell abnormal chromatin clamping* |
| Megaloblastoid change |
| Giant red cell* |
| Vacuolization |
| Howell -Jolly bodies* |
| Dysgranulopoiesis |
| Small size or unusually large size |
| Nuclear hyposegmentation (Psudo-Pelger–Huët) |
| Nuclear hypersegmentation |
| Decreased granules; agranularity |
| Pseudo-Chédiak–Higashi granules |
| Döhle bodies |
| Auer rods |
| Dysplastic non-pPH* |
| Nuclear projections (NP)* |
| Abnormal chromatin clumping* |
| Dysmegakaryopoiesis |
| Micromegakaryocytes |
| Nuclear hypolobation |
| Multinucleation |
| Large megakaryocyte with a hyperlobulated nucleus* |
| Megakaryocytes with cytoplasmic abnormality* |
| vacuolation, agranular or hypogranular cytoplasm, Persisting basophilia (nucleocytoplasmic asynchrony) |
| * Not included in World Health Organisation 2017 classification but in the International Working Group on Morphology of Myelodysplastic Syndrome classification.  Ring sideroblasts and periodic acid–Schiff (PAS) positivity in WHO 2017 classification were excluded in this study because special staining is required to assess these dysplasia. |

Supplemental Table 2. Prediction by network trained from scratch vs. True label

|  |  | Prediction | | | |
| --- | --- | --- | --- | --- | --- |
|  |  | DG0 | DG1 | DG2 | DG3 |
| True label | DG0 | 1565 | 78 | 18 | 0 |
|  | DG1 | 4 | 20 | 20 | 2 |
|  | DG2 | 8 | 24 | 42 | 3 |
|  | DG3 | 0 | 5 | 5 | 1 |
